# Antithrombin deficiency is associated with mortality and impaired organ function in septic pediatric patients: a retrospective study

**DOI:** 10.7717/peerj.5538

**Published:** 2018-09-05

**Authors:** Christian Niederwanger, Tobias Hell, Sophie Hofer, Christina Salvador, Miriam Michel, Bettina Schenk, Benedikt Treml, Mirjam Bachler

**Affiliations:** 1 Department of Pediatrics, Pediatrics I, Intensive Care Unit, Medical University of Innsbruck, Innsbruck, Austria; 2 Department of Mathematics, Faculty of Mathematics, Computer Science and Physics, University of Innsbruck, Innsbruck, Austria; 3 Department of General and Surgical Critical Care Medicine, Medical University of Innsbruck, Innsbruck, Austria; 4 Department of Pediatrics, Pediatrics I, Haematology and Oncology, Medical University of Innsbruck, Innsbruck, Austria; 5 Department of Pediatrics, Pediatrics III, Cardiology, Medical University of Innsbruck, Innsbruck, Austria; 6 Department of Sports Medicine, Alpine Medicine and Health Tourism, UMIT - University for Health Sciences, Medical Informatics and Technology, Hall in Tirol, Austria

**Keywords:** Children, Antithrombin, Mortality, Sepsis, Organ failure, C-reactive protein, Threshold level

## Abstract

**Background:**

Sepsis remains a major problem in intensive care medicine. It is often accompanied by coagulopathies, leading to thrombotic occlusion of small vessels with subsequent organ damage and even fatal multi-organ failure. Prediction of the clinical course and outcome—especially in the heterogeneous group of pediatric patients—is difficult. Antithrombin, as an endogenous anticoagulant enzyme with anti-inflammatory properties, plays a central role in controling coagulation and infections. We investigated the relationship between antithrombin levels and organ failure as well as mortality in pediatric patients with sepsis.

**Methods:**

Data from 164 patients under the age of 18, diagnosed with sepsis, were retrospectively reviewed. Antithrombin levels were recorded three days before to three days after peak C-reactive protein to correlate antithrombin levels with inflammatory activity. Using the concept of developmental haemostasis, patients were divided into groups <1 yr and ≥1 yr of age.

**Results:**

In both age groups, survivors had significantly higher levels of antithrombin than did deceased patients. An optimal threshold level for antithrombin was calculated by ROC analysis for survival: 41.5% (<1 yr) and 67.5% (≥1 yr). The mortality rate above this level was 3.3% (<1 yr) and 9.5% (≥1 yr), and below this level 41.7% (<1 yr) and 32.2% (≥1 yr); OR 18.8 (1.74 to 1005.02), *p* = 0.0047, and OR 4.46 (1.54 to 14.89), *p* = 0.003. In children <1 yr with antithrombin levels <41.5% the rate of respiratory failure (66.7%) was significantly higher than in patients with antithrombin levels above this threshold level (23.3%), OR 6.23 (1.23 to 37.81), *p* = 0.0132. In children ≥1 yr, both liver failure (20.3% vs 1.6%, OR 15.55 (2.16 to 685.01), *p* = 0.0008) and a dysfunctional intestinal tract (16.9% vs 4.8%, OR 4.04 (0.97 to 24.08), *p* = 0.0395) occurred more frequently above the antithrombin threshold level of 67.5%.

**Conclusion:**

In pediatric septic patients, significantly increased mortality and levels of organ failure were found below an age-dependent antithrombin threshold level. Antithrombin could be useful as a prognostic marker for survival and occurrence of organ failure in pediatric sepsis.

## Introduction

Despite considerable progress in treatment, sepsis is still associated with a high mortality rate and is the leading cause of death in patients with infectious diseases (Vincent et al., 2006; Angus et al., 2001). In children, it is one of the main causes of mortality and morbidity (Schlapbach et al., 2015; Boeddha et al., 2018; Hazelzet et al., 1996; Despond et al., 2001) and the most common cause of death in children under 5 years of age (Rudan et al., 2008).

Sepsis initiates diffuse activation of the coagulation system and at the same time inactivates anticoagulation as well as fibrinolysis. In its maximal variant, coagulation activity develops up to an uncontrolled coagulation process of disseminated intravascular coagulation (DIC). It leads to the formation of small intravascular blood clots that clog vessels and prevent the organs from being sufficiently supplied with blood, thus causing lasting damage to the organs. This condition is referred to as Multiple Organ Dysfunction Syndrome (MODS) (Schouten et al., 2008; Nimah & Brilli, 2003).

Biomarkers are needed to predict mortality and organ failure with high sensitivity and specificity in terms of diagnosis and prognosis. Due to the close correlation between coagulation and inflammation, coagulation markers are suitable for this purpose.

Septic patients usually have very low levels of antithrombin as antithrombin is increasingly consumed as a result of uncontrolled effluent coagulation in accordance with progress of sepsis (Mihajlovic et al., 2017). In many cases, synthesis is also impaired because of decreased liver performance in sepsis, and elastase released from activated neutrophils also inactivates antithrombin, a process promoted by heparin (Jordan, Kilpatrick & Nelson, 1987). Beside neutrophil elastase, syndecan shedding is another reason for the decrease in antithrombin (Chung et al., 2008).

An acquired antithrombin deficiency causes a dangerous imbalance in the coagulation system (Hayakawa et al., 2018). Antithrombin is not only an indispensable physiological anticoagulant, but also has anti-inflammatory properties—independent of its anticoagulant activity.

Although studies in adult patients report on the association between antithrombin and outcome during sepsis (Mihajlovic et al., 2017; Hayakawa et al., 2018), there is little literature available on children broken down into age groups (Ersoy et al., 2007; Hazelzet et al., 1996).

In particular, the relationship between antithrombin levels and the failure of various organs in the context of sepsis in children is poorly understood. Neither in adults nor in infants has the correlation between antithrombin and an organ failure of the gastrointestinal tract really been studied so far. No biomarkers have yet been confirmed for the diagnosis of Acute Respiratory Distress Syndrome (ARDS) or prediction of its prognosis (Garcia-Laorden et al., 2017; Cartin-Ceba et al., 2015). There may be an association with decreased antithrombin levels and acute liver failure at least in adult patients with end-stage heart failure (Hoefer et al., 2017).

In general, younger patients have physiologically lower levels of procoagulant factors as well as lower levels of fibrinolytic proteins (Attard et al., 2013; Jaffray & Young, 2013; Andrew et al., 1992). Coagulase inhibitors such as protein C, protein S and antithrombin are also reduced (Appel et al., 2012). While protein C and protein S remain reduced by 10%–20% of adult levels in childhood (Attard et al., 2013; Monagle et al., 2006; Andrew et al., 1988), antithrombin reaches adult levels only after 7–12 months (Appel et al., 2012).

Because antithrombin plays such an important role in sepsis, it may also be used as a predictive parameter for clinical outcome. In view of the much-discussed antithrombin administration with still unclear clinical benefit (Allingstrup et al., 2016), the question of threshold levels arises for a possible substitution trigger. Similar studies have found a threshold for antithrombin, but mainly for the adult (Pettila et al., 2002; Iba et al., 2015) or neonatal (Ersoy et al., 2007) patient population. The knowledge concerning our targeted pediatric patient population is poor, with especially a lack of recent studies. The aim of this study was to examine whether children with sepsis have a threshold level for antithrombin activity, at which antithrombin deficiency increases the probability of a negative outcome in terms of mortality and organ failure.

## Methods

Patients aged 0–18 years treated at the Pediatric Intensive Care Unit (PICU) of Innsbruck Medical University Hospital between January 2000 and December 2014 were screened for suspicious or proven infections. A total of 250 patients met the sepsis criteria of the international definitions for pediatric sepsis and organ dysfunction of 2005 (Goldstein, Giroir & Randolph, 2005). Furthermore, the children had to meet the inclusion criterion of an available antithrombin measurement at the peak level of C-reactive protein during sepsis. Finally, 164 pediatric patients were included in this retrospective analysis. Clinical data as well as the routine laboratory parameters C-reactive protein (CRP) and antithrombin (AT) levels were recorded for these patients. The study was permitted by the institutional review board of the Medical University of Innsbruck (AN2013-0044).

We collected the demographic variables age, sex, and the diagnosed underlying disease of the children. Characteristics of patients are listed in Table 1. We screened the children’s hospital stay for the day with the most severe C-reactive protein rash to observe the most severe stage of sepsis in every child, regardless of the underlying disease or any received sepsis treatement. The peak level of C-reactive protein was defined as day 0 and was used to objectify sepsis progression (Povoa et al., 2005; Schmit & Vincent, 2008). Due to a possible temporal displacement of the C-reactive protein peaks and the sepsis maximum, the available antithrombin levels were observed from three days before until three days after day 0 (C-reactive protein peak).

**Table 1:** Comparison of patient characteristics in children younger than 1 year (<1 year) and older than 1 year (≥1 year).

**Characteristics**^a^	**Total** (*n* = 164)	**<1 year** (*n* = 42)	≥1 year (*n* = 122)	**Estimate with 95% CI**^b^	*p* value^c^
Female gender	72/164 (43.9%)	19/42 (45.2%)	53/122 (43.4%)	1.07 (0.5 to 2.31)	0.8586
Age (months)	41.95 (9.72–134.6)	1.87 (0.78–4.32)	77.98 (34.22–163.19)	75.33 (49.5 to 105.03)	<0.0001
PIM2^d^ predicted mortality (%)	3.9 (1.1–8.3)	3.8 (1.1–15.1)	3.9 (1.1–7.2)	−0.2 (−2.3 to 0.9)	0.6229
**Diagnosed underlying disease**					
Central nervous system	35/164 (21.3%)	8/42 (19%)	27/122 (22.1%)	0.83 (0.3 to 2.11)	0.8278
Cardiovascular system	30/164 (18.3%)	16/42 (38.1%)	14/122 (11.5%)	4.69 (1.89 to 11.89)	0.0003
Digestive tract	29/164 (17.7%)	17/42 (40.5%)	12/122 (9.8%)	6.14 (2.43 to 16.11)	<0.0001
Respiratory system	40/164 (23.8%)	15/42 (35.7%)	25/122 (20.5%)	2.14 (0.92 to 4.94)	0.0608
Oncologic	25/164 (15.2%)	2/42 (4.8%)	23/122 (18.9%)	0.22 (0.02 to 0.95)	0.0269
Kidney	21/164 (12.8%)	5/42 (11.9%)	16/122 (13.1%)	0.9 (0.24 to 2.8)	1
Liver	15/164 (9.1%)	5/42 (11.9%)	10/122 (8.2%)	1.51 (0.38 to 5.23)	0.5362
Skin	6/164 (3.7%)	0/42 (0%)	6/122 (4.9%)	0 (0 to 2.46)	0.3396

**Notes.**

a Binary data are presented as no./total no. (%), continuous data as medians (25th–75th percentile).

b Odds ratio for binary variables and estimated median difference for continuous variables.

c Differences between groups assessed with Fisher’s exact test for binary variables and Wilcoxon rank sum test for continuous variables.

d Pediatric Index of Mortality Score 2 (not known for nine patients younger than 1 year and six patients older than 1 year).

The level of C-reactive protein was measured with an immunological turbidity test (Roche cobas^®^ system) and for the antithrombin level a functional chromogenic assay (Siemens Berichrom^®^ Antithrombin III (A) on Siemens BCS XP) was used (Price et al., 1987; Eda et al., 1998; Friberger et al., 1982).

Based on the fact that antithrombin activity reaches the adult level at the age of 7–12 months (Andrew et al., 1987; Andrew et al., 1992), patients were classified in age groups of <1 yr (younger than one year) and ≥1 yr (one year or older). Organ failure and in-hospital mortality were chosen as outcome parameters.

### Statistical analysis

A mathematician not involved in the study procedures or patient assessment was responsible for the statistical analyses using R version 3.4.1 (R Core Team, 2017). All statistical assessments were two-sided and a significance level of 5% was used. The hypothesis of a normal distribution was not reasonable for most of the continuous variables (Shapiro–Wilk normality test). To rule out possible confounding due to the protracted study period (2000–2014), we grouped patients into four time-cohorts: 54 patients had their C-reactive protein peak in 2000–2004, 35 in 2005–2007, 47 in 2008–2010 and 28 in 2011–2014. The Kruskal–Wallis test was used to look for differences between the time cohorts and also between the antithrombin levels stratified by underlying disease. The Wilcoxon rank sum test and Fisher’s exact test were applied to assess differences between the two age groups and between patients with antithrombin levels above and below the computed threshold.

We present continuous data as medians (25th–75th percentile) and binary variables as no./total no. (%). We show effect size or precision with estimated median differences between groups for continuous data and odds ratios (OR) for binary variables, with 95% CIs.

We perform a ROC curve analysis for survival predicted by antithrombin levels at the peak level of C-reactive protein to compute optimal thresholds for antithrombin. For children older than one year and stratified according to survival, the evolution antithrombin levels from three days prior to three days after the peak level of C-reactive protein are illustrated by the sequence of medians with corresponding 95% CIs in a purely descriptive manner.

## Results

A total of 164 critically ill children with an available antithrombin measurement at the time of peak level of C-reactive protein were included in the final analysis. For patient characteristics see Table 1. The children older than one year had a median age of 6.5 years, whereas those younger than 1 year were median 1.9 months old. Predicted mortality in both groups was comparable.

In children <1 yr significantly more organs were affected than in children ≥1 yr. Children aged less than 1 year most commonly showed an underlying disease in the form of disorders of the digestive tract (40.5%) followed by cardiovascular complications (38.1%) and problems of the respiratory system (35.7%). Children older than 1 year most often presented with disorders of the central nervous system (22.1%), followed by complications of the respiratory system (19.7%) and oncologic diseases (18.9%).

**Figure 1:**
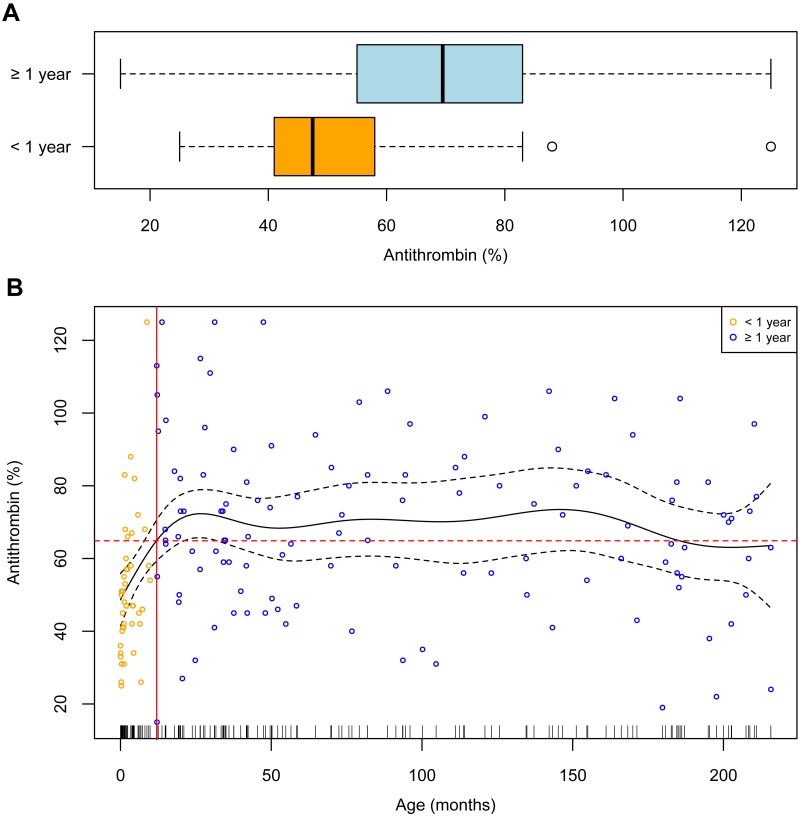
Relationship between antithrombin levels and age. (A) Boxplots of antithrombin levels for patients younger (<1 year) and older than one year (≥1 year). (B) Smoothing spline (black solid line) with 95% CI (dashed black lines). The solid red line marks 12 months of age and the dashed red line corresponds to the mean antithrombin level of 64.87%.

As depicted in Fig. 1A, antithrombin levels at the peak level of C-reactive protein were significantly lower in children <1 yr, namely 47.5 (41–58), than in children ≥1 yr, 69.5 (55.25–83); estimated median difference 19 (12 to 26), *p* < 0.0001. In children ≥1 yr the antithrombin levels were systematically higher, as seen in Fig. 1B. For progression analysis in children <1 yr, too few antithrombin measurements were available. Therefore, the analysis is restricted to day 0 in this patient group.

To evaluate the protracted study period (2000–2014), we grouped patients into four time-cohorts: 54 patients had their C-reactive protein peak in 2000–2004, 35 in 2005–2007, 47 in 2008–2010 and 28 in 2011–2014. The mortality rate was 13.0%, 31.4%, 14.9% and 21.4%, respectively, and was not significantly associated with the time periods (Fisher’s Exact test: *p* = 0.1527). Median Paediatric Index of Mortality, Version 2 (PIM2), scores were accordingly higher in time periods with higher mortality: 3.3 (1.1–5.9) in 2000–2004, 4.35 (1.3–14.95) in 2005–2007, 2.05 (0.92–6.6) in 2008–2010 and 5.9 (3.9–13.9) in 2011–2014 (Kruskal–Wallis test: *p* = 0.0324). Moreover, C-reactive protein levels and antithrombin levels did not significantly differ between time cohorts (Kruskal–Wallis test: *p* = 0.1642 and *p* = 0.2437, respectivley).

### Antithrombin threshold levels for survival

**Figure 2:**
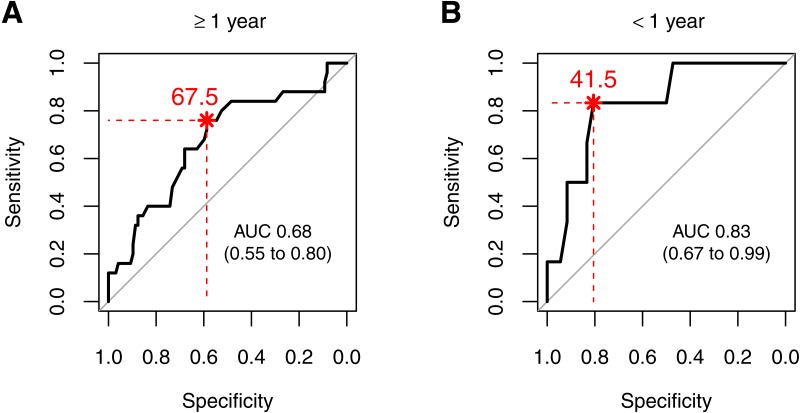
ROC curves for survival predicted by antithrombin levels for patients (A) older than one year (≥1 year) and (B) younger than one year (<1 year). The optimal threshold for antithrombin levels (%) are written in red text and corresponds to the point closest to the asterisk.

Statistical significance for survival depending on antithrombin was found in both age groups when calculating a threshold level. An antithrombin level above 67.5% was associated with a better outcome in children ≥1 yr, whereas in younger children an antithrombin threshold level of 41.5% was associated with an increased survival rate (Fig. 2). Due to the heterogeneity of patients the antithrombin levels were tested for differences between the groups of underlying diseases. This resulted in significantly not different antithrombin levels in both age classes, children younger than one year (*p* = 0.7614) and older than one year (*p* = 0.1309).

**Figure 3:**
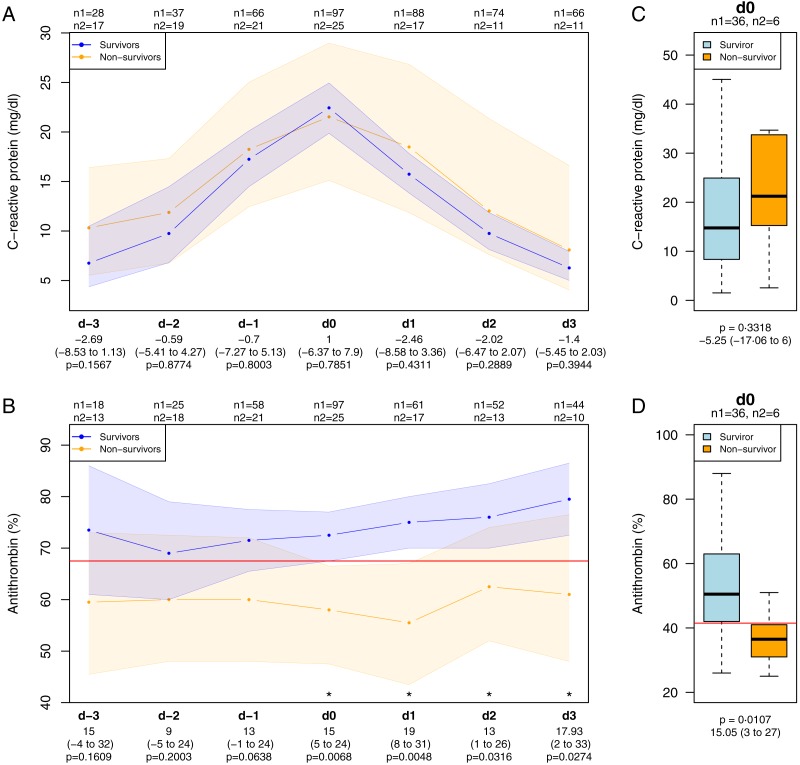
C-reactive protein and antithrombin levels in children with sepsis. Progression of C-reactive protein levels (A) and antithrombin levels (B) in children older than one year stratified by survival. Depicted are medians with 95% CIs. Boxplots of C-reactive protein levels (C) and antithrombin levels (D) for patients younger than one year at the peak levels of C-reactive protein. The red lines refer to the computed threshold levels for antithrombin. n1, children who survived; n2, children who deceased. d-3 to d3 describe the days of the observation period whereas d0 is the day of C-reactive protein peak level, starting with d-3 three days before and ending with d3 three days after d0.

In children ≥1 yr, progression of C-reactive protein during the observation period did not discriminate between survivors and non-survivors, whereas the antithrombin levels were significantly different in survivors and non-survivors from day 0, as depicted in Figs. 3A and 3B). Similarly, in children <1 yr the C-reactive protein levels did not differ between survivors and non-survivors at day 0, but antithrombin did (Figs. 3C and 3D).

Of the children <1 yr with respiratory failure 66.7% showed antithrombin levels below the threshold of 41.5%. Of the older children with liver failure 20.3% and of those with complications of the intestinal tract 16.9% revealed antithrombin levels lower than the calculated threshold of 67.5%. Other single organ failures and the multiple organ dysfunction syndrome were evenly distributed in dependence on antithrombin threshold levels (Table 2).

**Table 2:** Children’s morbidity stratified by age and antithrombin (AT) levels (%).

**Morbidity**^a^	**Children <1 year**					**Children**≥**1 year**				
	Total (*n* = 42)	AT ≥41.5 (*n* = 30)	AT<41.5 (*n* = 12)	OR with 95% CI^b^	*p* value^c^	Total (*n* = 122)	AT ≥67.5 (*n* = 63)	AT<67.5 (*n* = 59)	OR with 95% CI^b^	***p* value**^c^
Survivor	36/42 (85.7%)	29/30 (96.7%)	7/12 (58.3%)	18.8 (1.74 to 1005.02)	0.0047	97/122 (79.5%)	57/63 (90.5%)	40/59 (67.8%)	4.46 (1.54 to 14.89)	0.003
**Diagnosed organ failure**										
Cardiovascular system	16/42 (38.1%)	10/30 (33.3%)	6/12 (50%)	1.97 (0.41 to 9.63)	0.483	21/122 (17.2%)	9/63 (14.3%)	12/59 (20.3%)	1.53 (0.54 to 4.5)	0.4734
Central nervous system	2/42 (4.8%)	1/30 (3.3%)	1/12 (8.3%)	2.57 (0.03 to 213.54)	0.4948	10/122 (8.2%)	3/63 (4.8%)	7/59 (11.9%)	2.67 (0.57 to 16.82)	0.1951
Intestinal tract	9/42 (21.4%)	6/30 (20%)	3/12 (25%)	1.32 (0.18 to 7.98)	0.6987	13/122 (10.7%)	3/63 (4.8%)	10/59 (16.9%)	4.04 (0.97 to 24.08)	0.0395
Kidney	6/42 (14.3%)	4/30 (13.3%)	2/12 (16.7%)	1.29 (0.1 to 10.78)	1	28/122 (23%)	14/63 (22.2%)	14/59 (23.7%)	1.09 (0.43 to 2.77)	1
Liver	3/42 (7.1%)	3/30 (10%)	0/12 (0%)	0 (0 to 6.15)	0.5453	13/122 (10.7%)	1/63 (1.6%)	12/59 (20.3%)	15.55 (2.16 to 685.01)	0.0008
Respiratory	15/42 (35.7%)	7/30 (23.3%)	8/12 (66.7%)	6.23 (1.23 to 37.81)	0.0132	28/122 (23%)	13/63 (20.6%)	15/59 (25.4%)	1.31 (0.52 to 3.35)	0.6671
Multiple organ dysfunction syndrome	22/42 (52.4%)	15/30 (50%)	7/12 (58.3%)	1.39 (0.3 to 6.92)	0.7385	46/122 (37.7%)	22/63 (34.9%)	24/59 (40.7%)	1.28 (0.58 to 2.84)	0.5768
Thromboembolic event	5/42 (11.9%)	2/30 (6.7%)	3/12 (25%)	4.46 (0.44 to 61.51)	0.1309	4/122 (3.3%)	2/63 (3.2%)	2/59 (3.4%)	1.07 (0.08 to 15.21)	1
Bleeding event	2/42 (4.8%)	1/30 (3.3%)	1/12 (8.3%)	2.57 (0.03 to 213.54)	0.4948	5/122 (4.1%)	1/63 (1.6%)	4/59 (6.8%)	4.46 (0.42 to 225.31)	0.1964

**Notes.**

a Data are presented as no./total no. (%).

b Odds ratio for binary variables.

c Differences between groups assessed with Fisher’s exact test.

## Discussion

Sepsis with concomitant disruption of the coagulation system up to DIC and the associated consumption of coagulation factors lead to a condition of acquired antithrombin deficiency (Amaral, Opal & Vincent, 2004). It can be deduced that the level of antithrombin is related to the severity of the coagulation disorder and consequently affects the extent of organ damage and survival (Fourrier et al., 1992). The aim of this study was to investigate the relationship between antithrombin activity and the likelihood of survival and organ damage in pediatric patients with sepsis. The significantly lower antithrombin levels in the age group <1 yr than in the age group ≥1 yr confirm the concept of developmental hemostasis introduced by Andrew et al. (1987); Andrew et al. (1992). In studies conducted in septic adult patients, an association between increased mortality and low antithrombin levels of around 63% has been demonstated (LaRosa et al., 2006; Taylor Jr et al., 1988). The Kypercept trial revealed that in patients with antithrombin levels <60% the mortality rate was elevated to up to 47.5% in comparison to patients with a higher antithrombin level, where the mortality rate was increased to up to 29.1% (Warren et al., 2001). In our study a threshold level of 67.5% was calculated, which discriminates between survivors and non-survivors on the day of C-reactive protein peak in children older than one year. The lower threshold value, namely an antithrombin level of 41.5% in children <1 yr, is in line with other studies: comparable levels of antithrombin (52.0%) were found in neonates with suspected sepsis (Bartolovic et al., 2011), and also in this patient group the survivors had higher antithrombin levels (Ersoy et al., 2007; Lauterbach et al., 2006).

C-reactive protein levels did not differ between survivors and non-survivors in either age group, although C-reactive protein was shown to be of diagnostic value in sepsis in other studies (Pasternak et al., 2016; Lautz et al., 2016; Carcillo et al., 2017).

In our study, it was not only possible to statistically differentiate between survival and non-survival (*p* = 0.003) based on the antithrombin level in the age group ≥1 yr, but also to determine whether a failure of certain organs occurs or not. In the context of sepsis, microangiopathy caused by disruption of clotting activity can lead to impairment of organ functions including organ failure (Amaral, Opal & Vincent, 2004). The highest statistical relevance was observed in the prediction of liver failure (*p* = 0.0008) in the patient group ≥1 yr, which in children with antithrombin deficiency occurred at a significantly higher frequency of more than 20%.

The association between impaired liver function and low antithrombin levels, which was clearly demonstrated in this study, can be explained by a pre-existing liver failure and the resulting reduced antithrombin production (Sheikh Sajjadieh & Vasilovna Viunytska, 2009). On the other hand, this can also be attributed to DIC-induced hypoperfusion of the liver in the context of sepsis. Antithrombin not only optimizes coagulation, but is also a potential regulator of inflammatory processes and subsequent tissue damage. Injection of antithrombin directly into the portal vein after LPS-induced acute liver failure resulted in a significant reduction in inflammatory cytokines, reduced intrahepatic fibrin deposition and improvement of the histological findings (Miyazaki et al., 2012). In a rat model, systemic administration of antithrombin was seen to improve liver function in liver failure and attenuate damage of the liver tissue in a dose-dependent manner (Fujiwara et al., 1988). As an underlying mechanism increased anithrombin-intitated prostacyclin distribution was suspected (Harada et al., 1999), which might have been the reason for inhibition of platelet aggregation and reduction of thrombocytopenia (Fujiwara et al., 1988). In adult cancer patients, antithrombin levels of <50%–61.5% predicted postoperative liver dysfunction (Pereyra et al., 2017; Hoffmann et al., 2006), and administration of antithrombin was able to reduce this rate (Kuroda et al., 2015).

In the age group ≥1 yr, a significant correlation (*p* = 0.0395) was found between lower antithrombin and increased rate of organ damage of the gastrointestinal tract (16.9%). So far, there are hardly any studies that describe this effect between antithrombin and organ failure of the gastrointestinal tract in humans. In rat models antithrombin had a positive effect on reperfusion after intestinal injury due to a reduction in fibrin deposition and micro-vascular thrombotic obstruction as well as anti-inflammatory action (Schoots et al., 2004). Furthermore, decreased leukocyte migration and adhesion along mesenterial venoles after endotoxin treatment was proven (Neviere et al., 2001). The reason was the inhibition of thrombin action on the endothelium, resulting in the expression of adhesion molecules (Ostrovsky et al., 1997). Consequntly, antithrombin is able to reduce organ tissue damage and subsequent failure.

In the patient group <1 yr, low antithrombin levels were associated with an increased rate of respiratory failure (*p* = 0.0132). Like other organs, the lung is affected by DIC and inflammatory reactions in sepsis. Several studies suggested that low levels of antithrombin are combined with poor outcomes in lung disease such as acute lung injury (ALI) and idiopathic respiratory distress syndrome (IRDS) in neonates (Peters et al., 1984; Van den Berg et al., 1989) and adults (Owings & Gosselin, 1997). Again, in rat models less tissue destruction of the pulmonary vessels was detected after antithrombin administration following LPS-induced sepsis, which was attributed to the ability of antithrombin to induce prostacyclin release and thereby reduce leukocyte activation (Uchiba & Okajima, 1997). In another rat model treatment of pneumonia, triggered by S. pneumoniae, with antithrombin resulted in a marked reduction in neutrophil cells in the lung, decreased levels of pro-inflammatory cytokines, and a reduction in NET (neutrophil extracellular trap) formation (Ishikawa et al., 2017; Choi et al., 2008). Here, too, a reduction in lung tissue destruction as well as a significant reduction in colony formation of S. pneumoniae was demonstrated (Choi et al., 2008). This leads us to conclude that by interacting with the complexity of an inflammatory reaction antithrombin exerts a certain protective effect on the lung in the context of sepsis.

Surprisingly, we found no association between low levels of antithrombin and kidney failure or underlying kidney disease. Other studies in adults have clearly shown that antithrombin may help limit acute kidney injury (Yin et al., 2017; Kong et al., 2017; Wang et al., 2015; Rameshkumar et al., 2017).

Although not significantly different, more bleeding complications as well as thromboembolic events are observed in children with antithrombin levels below the calculated threshold levels, especially in children younger than 1 year.

Although the results suggest that higher levels of antithrombin are associated with a better outcome, the data do not suggest that antithrombin as a drug also improves the outcome of sepsis patients. Early studies in human populations showed controversial effects; the benefit of antithrombin in the treatment of critically ill patients remained unclear (Fourrier et al., 1993; Baudo et al., 1998; Inthorn et al., 1998; Eisele et al., 1998; Gando et al., 2013; Hoffmann et al., 2004). The Kypersept trial was not able to prove a survival benefit, but even caused higher bleeding rates in septic adults receiving antithrombin substitution (Warren et al., 2001). Nevertheless, critical analysis of this study showed a clear tendency (*p* = 0.058) to a lower rate of new organ failure after treatment in the antithrombin group as compared to the placebo group (Eid, Wiedermann & Kinasewitz, 2008). Moreover, the mortality rate significantly decreased in patients with antithrombin administration, namely from 44.9% in the placebo group to 52.5% when not concomitantly receiving heparin (Hoffmann et al., 2006).

Our study design does not allow a conclusion about a trigger limit with respect to administration. However, our levels of safekeeping certainly indicate a critical threshold of antithrombin activity, which may possibly be used as a therapy-critical basis.

Compared to adults, only few studies of antithrombin substitution have been conducted in children. These also report conflicting results regarding efficacy and safety (Kreuz, Schneider & Nowak-Gottl, 1999; St Peter et al., 2007; Wong et al., 2016), but the administration of antithrombin in children is increasing (Wong et al., 2013). Especially in infants, antithrombin administration showed an improvement in multiple-organ dysfunction including an increase in platelet count with a good safety profile (Novakova et al., 2000).

Patients in this study did not receive antithrombin rountinely, but we were not able to discriminate patients who had been administered antithrombin from those who had not. Even if both production and consumption are certainly involved in the pathophysiologic pathways of coagulation, especially the aspects of liver synthesis on the one hand and coagulation on the other hand, as prognostic factors should be examined in future studies focusing on differences between substituted and non-substituted antithrombin levels.

In this study, patients were included over a 14-year period. The increased mortality in the children in the period from 2005–2007 is probably attributed to the admission of initially more severely ill patients and not to to a change in practice during this time.

Our study demonstrates that a low antithrombin level in septic children is a good prognostic marker for certain organ failures depending on the age group and is associated with higher mortality throughout childhood. If antithrombin supplementation reaches levels above the calculated thresholds, the improving organ function and mortality rate need to be evaluated in further studies.

### Limitations

An important limitation of our study is the heterogeneity of the patients regarding the different underlying diseases. Unfortunately, the sample size was too small to calculate threshold levels for each affected system, especially in oncologic patients, which is difficult to achieve since the overall number of critically ill children is low. Nevertheless, there was no difference in antithrombin levels regarding the underlying diseases in our patient population.

Another confounding factor is that treatments, e.g., antithrombin supplementation were not taken into account in this analysis and so could have influenced the levels of both C-reactive protein and antithrombin. C-reactive protein was used only as a surrogate marker in order to identify the most severe period of the sepsis in each child independently of any treatment; the absolute levels of C-reactive protein are secondary for the objective of this study. Furthermore, we assumed in this study that administered antithrombin has the same efficacy as endogenous antithrombin and therefore no discrimination was necessary.

Due to the reaction time and the limited duration of the elevated serum level, the C-reactive protein is also unsuitable for immediate diagnosis and prognosis (Haupt et al., 1996; Martin et al., 1997). Thus, the C-reactive protein peak does not coincide exactly with the maximum temporal manifestation of sepsis but is close to it.

Nonetheless, the antithrombin threshhold levels for survival and organ failure should be analysed in a larger patient cohort stratified according to underlying disease since the levels could be influenced by these illnesses.

Unfortunately, the study design does not allow the evolution of sepsis to be observed from the beginning since the study center is a high-level PICU and many children were transferred to our hospital when sepsis had already progressed. This also explains the high mortality since our PICU treats the most severe cases of sepsis.

## Conclusion

Children with sepsis revealed age-dependent antithrombin threshold levels, below which dysfunction of particular organs and mortality significantly increased. At an antithrombin level of 41.5% the threshold level for significantly higher morbidity and mortality was lower in children <1 yr than it was in children ≥1 yr, with a threshold value of 67.5%. Lower antithrombin levels may be seen as a prognostic tool for increased morbidity and mortality in pediatric sepsis patients.

##  Supplemental Information

10.7717/peerj.5538/supp-1 File S1 Antithrombin levels stratified by underlying disease. Boxplots of antithrombin levels (%) by underlying diseases of (A) children older (≥1 year) and (B) younger than one year (<1 year). CNS refers to underlying diseases affecting the central nervous system.

10.7717/peerj.5538/supp-2 File S2 Raw data Data set of antithrombin in septic children

## Abbreviations

<1 yr: younger than one year
≥1 yr: one year or older
PIM2: Pediatric Mortality Index, Version 2
AT: antithrombin
CRP: C-reactive protein

## Additional Information and Declarations

### Data Availability

The following information was supplied regarding data availability: The raw dataset is provided as a Supplemental File.
