# Supplementary figures and images for: Antithrombin deficiency is associated with mortality and impaired organ function in septic pediatric patients: a retrospective study
Source: PeerJ. 2018 Sep 5;6:e5538. doi: 10.7717/peerj.5538 (PMC6129139; doi:10.7717/peerj.5538)

**A** $\geq 1$  year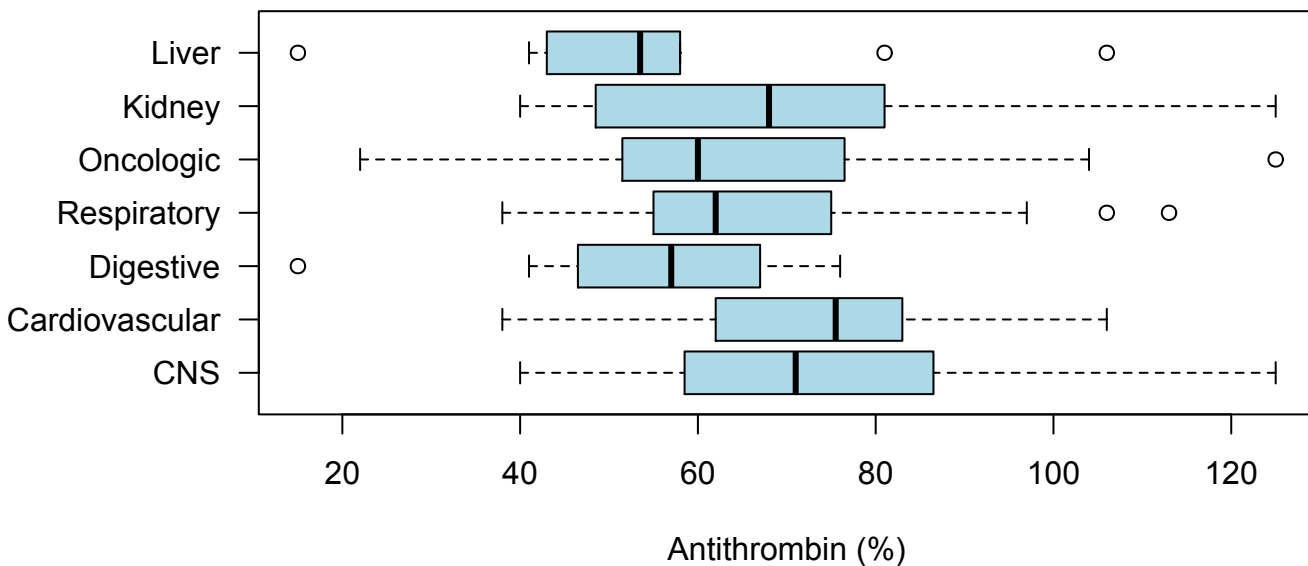**B** $< 1$  year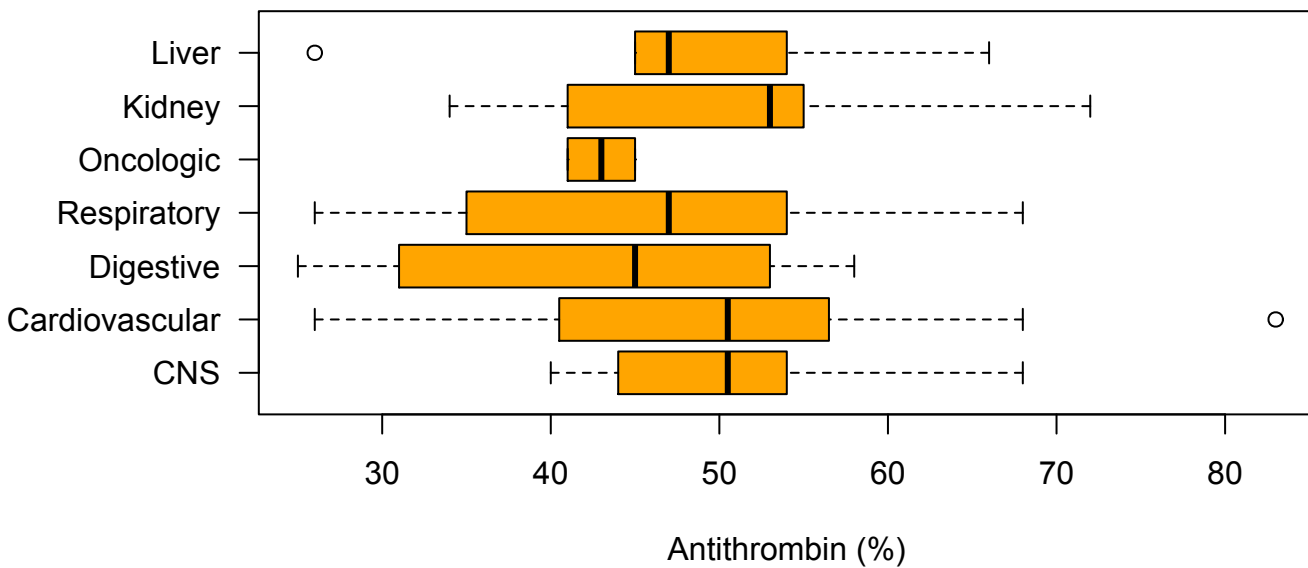

Supplement: File S1 — Boxplots of antithrombin levels (%) by underlying diseases of (A) children older (≥1 year) and (B) younger than one year (<1 year). CNS refers to underlying diseases affecting the central nervous system. [file peerj-06-5538-s001.pdf]
